# Exosomal Non-Coding RNAs as Potential Biomarkers for Alzheimer’s Disease: Advances and Perspectives in Translational Research

**DOI:** 10.3390/ijms26178246

**Published:** 2025-08-25

**Authors:** Simoneide Souza Titze-de-Almeida, Clara Luna Marina, Milena Vieira Ramos, Letícia Dias dos Santos Silva, Pedro Renato de Paula Brandão, Diógenes Diego de Carvalho Bispo, Felipe Von Glehn, Ricardo Titze-de-Almeida

**Affiliations:** 1 Research Center for Major Themes—Neurodegenerative Disorders Division, University of Brasília, Brasília 70910-900, Federal District, Brazil; 2 Faculdade de Medicina, University of Rio Verde, Aparecida de Goiânia 74923-590, Goiás, Brazil; 3 Sírio-Libanês Hospital, Brasilia 70200-730, Federal District, Brazil; 4 Brasília University Hospital, University of Brasília, Brasília 70910-900, Federal District, Brazil

**Keywords:** Alzheimer’s disease, biomarkers, β-amyloid, tau protein, miRNAs, circRNAs

## Abstract

Alzheimer’s disease (AD) is a progressive neurodegenerative disorder primarily characterized by memory loss and cognitive decline, which significantly impacts patients’ quality of life and imposes substantial emotional, practical, and economic burdens on their families. As the most common cause of senile dementia, AD currently affects approximately 50 million people worldwide, with projections indicating a threefold increase by 2050 due to rising life expectancy and an aging global population. Diagnosis of AD remains challenging. Neuroimaging techniques reveal atrophy in critical brain regions, particularly in the cortex, hippocampus, and limbic system, which are essential substrates for memory, personality changes, and other cognitive functions. The hallmark molecular changes associated with AD include the accumulation of β-amyloid plaques and the formation of tau protein tangles. Several underlying mechanisms contribute to neuron loss, such as oxidative stress, neuroinflammation, microbial dysbiosis, and insulin resistance. In this context, exosomes—small extracellular vesicles that facilitate cell communication—transport proteins, DNA, mRNA, and non-coding RNA (ncRNA), all of which play a significant role in the neurobiology of AD. Furthermore, emerging research indicates that exosomal ncRNAs may serve as promising biomarkers for AD, offering the possibility of improved diagnostic precision. This review explores the potential of exosomal ncRNAs—specifically circular RNAs and microRNAS—as non-invasive biomarkers for AD, highlighting recent advances and future directions in translational studies.

## 1. Introduction

Dementia has increasingly become a focal point in scientific research and public health initiatives. This attention is driven by the condition’s rising prevalence among the aging global population, as well as its profound impact on both affected individuals and their families. Efforts are being made in the realms of prevention, early diagnosis, and clinical intervention to address this growing concern [1]. The increase in life expectancy has contributed to a rise in cases of dementia as well as other neurodegenerative disorders among the elderly. Currently, approximately 50 million individuals worldwide are diagnosed with dementia, a figure projected to triple by 2050 [2]. Alzheimer’s disease is the most common form of dementia globally. This chronic neurological disease was first described by Alois Alzheimer in 1906 and is primarily characterized by progressive memory loss and impairment across multiple cognitive functions [3]. Despite considerable progress in understanding the brain pathology associated with AD, diagnosing and treating this complex disorder remain challenging [1,4].

The incidence of AD is influenced by non-modifiable factors such as advancing age, female sex, lower educational attainment, and genetic predisposition, particularly the presence of the APOE (Apolipoprotein E) ε4 allele, while ε2 offers some protection. Modifiable factors include cardiovascular conditions (hypertension, diabetes, hypercholesterolemia), lifestyle elements (Mediterranean diet, physical activity, cognitive engagement), and environmental exposures (e.g., air pollution). Depression, obesity, subjective cognitive decline, neurodegenerative markers on imaging, and co-pathologies also contribute to AD risk [5,6,7,8,9,10,11,12,13].

The pathological features of AD encompass the accumulation of β-amyloid (Aβ) plaques and neurofibrillary tangles (NFTs) composed of tau protein within the brain. Additional changes of note include an increase in reactive oxygen species (ROS), the proliferation of glial cells, impaired insulin sensitivity, and modifications in the microbiome [14]. These characteristics have been extensively studied due to their critical importance for both diagnostic and potential therapeutic applications [15].

Exosomes are a type of extracellular vesicles (EVs) that have gained significant prominence in AD research in recent years. They are small lipid vesicles that carry a variety of molecules, including proteins, DNA, mRNA, and non-coding RNA (ncRNA) from their cells of origin. Notably, ncRNAs are regulators of gene expression and can influence multiple signaling pathways. The major types of ncRNAs include microRNAs (miRNAs), which regulate gene expression post-transcriptionally; long non-coding RNAs (lncRNAs), involved in transcriptional and post-transcriptional control; and circular RNAs (circRNAs) that can act as miRNA sponges. Other types, such as small nuclear RNAs (snRNAs), small nucleolar RNAs (snoRNAs), and PIWI-interacting RNAs (piRNAs), contribute to RNA processing and genome stability [16,17,18]. AD-related exosomes have been shown to disseminate amyloidogenic peptides, suggesting their involvement in the underlying mechanisms of brain pathology [19].

Research has also investigated blood-derived exosomes as potential biomarkers for AD, focusing on the levels of Aβ, amyloid precursor protein (APP) fragments, tau, and phosphorylated tau (p-tau) [16,17,20]. Furthermore, exosomes isolated from AD patients exhibit aberrant contents of synaptic proteins, inflammatory mediators, growth factors, and lysosomal proteins [20,21,22,23].

Exosomal ncRNAs, miRNAs, have emerged as key regulators of APP and tau proteins, both of which are critical in AD pathology [24]. In addition to miRNAs, exosomes transport a variety of other ncRNAs, including lncRNAs and circRNAs, which facilitate cell-to-cell communication and reciprocal influence [25,26]. The expression patterns of these exosomal ncRNAs can vary depending on cell type and disease state, suggesting their potential involvement in AD and other pathologies [27,28]. Recent studies have further clarified the roles of exosomal mRNAs, circRNAs, and miRNAs in AD progression, particularly their regulatory effects on APP and tau proteins [24,29,30].

Regarding the diagnosis of AD, it is crucial to develop simple and effective tests with high specificity to improve diagnostics [15,31,32]. Exosomes represent a promising source of accessible and cost-effective biomarkers for disease monitoring, as their lipid bilayer protects the cargo from enzymatic degradation in the bloodstream [27,28]. This review examines the potential of ncRNAs derived from exosomes as viable biomarkers for AD.

## 2. Clinical Diagnosis of AD

Alzheimer’s disease is clinically defined as a major or mild neurocognitive disorder (NCD), as outlined in the Diagnostic and Statistical Manual of Mental Disorders, Fifth Edition (DSM-5). The DSM-5 emphasizes a decline in cognitive functioning that is not attributable to delirium or another mental disorder. Key criteria include significant cognitive decline in one or more cognitive domains (e.g., executive function, learning and memory, complex attention, language, social cognition, or perceptual motor skills) based on concerns from the individual, informants, or clinicians, and preferably documented through standardized neuropsychological testing. For a major NCD, these deficits must interfere with independence in daily activities, while for a mild NCD, independence remains intact but may require compensatory strategies. A diagnosis of probable AD requires genetic evidence or specific patterns of cognitive decline and progression, whereas possible AD is diagnosed in the absence of such evidence but with clinical support [33,34,35].

Associated with this clinical presentation, AD is characterized neuropathologically by the presence of amyloid plaques and NFTs, alongside other pathological changes such as inflammatory responses, astrocyte and microglial activation, and synaptic and neuronal loss. Amyloid plaques are extracellular deposits of Aβ peptides that follow a predictable regional progression, starting in the neocortex and advancing to other brain areas, as outlined in Thal phases [36,37,38,39,40]. NFTs, on the other hand, consist of intracellular aggregates of p-tau, which progress in a stereotyped pattern from the transentorhinal region to the limbic system and eventually the neocortex, as described in Braak stages [41]. The National Institute on Aging-Alzheimer’s Association (NIA-AA) guidelines recommend a comprehensive neuropathological assessment using the ABC scoring system, which integrates Thal phases for amyloid plaques, Braak stages for NFTs, and the Consortium to Establish a Registry for Alzheimer’s Disease (CERAD) criteria for neuritic plaque density. This approach allows for a detailed classification of AD-related changes, while also accounting for co-pathologies, such as cerebral amyloid angiopathy and other proteinopathies, that may influence clinical presentation and disease progression [36,37,38,39,40].

Historically, the diagnosis of AD relied primarily on clinical assessment, making accurate diagnosis both challenging and time-consuming. Physicians needed to exclude other potential causes of dementia through a rigorous, multi-step process. This typically involved a comprehensive review of medical history, physical examinations, laboratory tests to exclude other conditions (e.g., vitamin B-12, syphilis serology, and thyroid hormone levels), imaging studies (e.g., computed tomography (CT) scans), and the discontinuation of any medications that might impair cognition. Only after systematically ruling out other causes of cognitive decline could a probable AD diagnosis be established, with confirmation gained by observing disease progression and specific cognitive patterns associated with AD [42]. When clinical findings are inconclusive, re-evaluation is often necessary [43]. Moreover, early diagnosis of AD is frequently impeded by social and emotional barriers, including denial, fear of the diagnosis, limited family support, and difficulties in accessing healthcare [44].

Neuropathological studies in individuals diagnosed with AD during their lifetime have significantly advanced our understanding of the disease’s complexity and heterogeneity. While the hallmark pathological features—amyloid plaques composed of Aβ deposits and NFTs of p-tau proteins—are central to AD diagnosis, not all individuals clinically diagnosed with AD exhibit these findings. Approximately 14% of patients with mild-to-moderate AD lack sufficient neuropathological changes to meet classical diagnostic thresholds, as highlighted in postmortem brain studies. Among clinically diagnosed AD cases, 81% show high or intermediate levels of typical AD pathology, yet only 41% of dementia cases are attributed exclusively to AD pathology. Polymorbidity is common, with frequent co-occurrence of vascular lesions, TDP-43 proteinopathy, and Lewy bodies, which can complicate clinical presentations. This heterogeneity is also reflected in atrophy-based subtypes, such as limbic-predominant and hippocampal-sparing patterns, which correlate with variations in amyloid and tau pathology. The predictable progression of AD pathology described in Thal and Braak staging is often accompanied by comorbidities that may modify disease trajectories and explain variability in treatment outcomes. These findings underscore the importance of integrating neuropathological, clinical, and imaging data to provide a comprehensive understanding of AD and related dementias [39,45,46,47,48].

The diagnosis of AD has undergone significant evolution, with recent developments highlighting the integration of biological and clinical criteria. In 2024, the Alzheimer’s Association (AA) workgroup, led by Clifford Jack, proposed revised criteria emphasizing the biological definition of AD, centered on the ATN model, which incorporates biomarkers for Aβ, tau, and neurodegeneration. These criteria distinguish between Core 1 biomarkers—including amyloid PET (Positron Emission Tomography—an advanced neuroimaging technique used in the diagnosis and research of AD), CSF measures (specifically CSF Aβ42/40, CSF p-tau181/Aβ42, CSF t-tau/Aβ42), and plasma markers like phosphorylated tau 217 (requiring at least 90% accuracy for diagnostic validity)—and Core 2 biomarkers, comprising tau PET and specific fluid T2 biomarkers (indicative of, or correlate with, T2-weighted MRI findings, such as white matter hyperintensities, which are common in neurodegenerative diseases) such as MTBR-tau243 (specific fragment of the tau protein that includes the microtubule-binding repeat (MTBR) domain and ends at amino acid 243) and other phosphorylated tau forms [49]. Core 1 biomarkers define the earliest in vivo detectable stage of AD and can identify disease presence in both symptomatic and asymptomatic individuals. In contrast, Core 2 biomarkers become abnormal later in disease evolution and are more closely linked to symptom onset, serving primarily for biological staging and prognosis rather than diagnosis. Under this framework, an abnormal Core 1 biomarker is sufficient for an AD diagnosis, even in the absence of clinical symptoms, driving the inclusion of patients in clinical trials of drugs targeting disease biology.

However, this approach has sparked debate in the scientific community. The conceptualization of AD as a clinical–biological construct has been a cornerstone of the International Working Group’s (IWG) approach, emphasizing the necessity of integrating specific clinical phenotypes with pathophysiological biomarkers. According to the IWG, the diagnosis of AD should rest on the presence of distinct clinical syndromes—such as the amnestic variant or other characteristic phenotypes—supported by positive biomarkers, ensuring that these biological findings are interpreted within a clinical context. This approach contrasts with the AA framework, which allows for the diagnosis of AD in cognitively normal individuals based solely on biomarker positivity [50]. The AA framework often assumes that the amyloid cascade exhibits full biological and clinical penetrance, with minimal influence from co-pathology or unknown protective factors. However, a recent study in the Alzheimer’s Disease Neuroimaging Initiative (ADNI) cohort found that only about 36% of participants conformed to the amyloid cascade, as determined by tau PET cutoff points. This suggests that only approximately one-third of amyloid-positive individuals in the ADNI population follow the expected trajectory outlined by the AA framework. Moreover, the presence of AD biomarkers does not fully explain clinical symptomatology in nearly two-thirds of cases. In low-penetrance scenarios, where population heterogeneity is significant, biomarkers have limited predictive power, and monoclonal antibody treatments may have minimal impact on disease progression [51].

A major point of contention lies in the classification of cognitively normal individuals with positive biomarkers. The AA criteria consider these individuals as having AD, while the IWG advocates for alternative terminology, categorizing them as “asymptomatic at risk for AD” or, in specific scenarios with deterministic biomarker profiles, as having “presymptomatic AD” [49,50]. The IWG highlights the potential risks of labeling cognitively normal individuals as having AD based solely on biomarkers, including psychological distress, social consequences, and the possibility of overdiagnosis. Moreover, they emphasize that many biomarker-positive individuals may never develop clinical symptoms, underscoring the importance of distinguishing risk states from active disease to avoid unnecessary interventions and anxiety [49,50,52].

Both frameworks acknowledge the importance of staging disease progression and recognize factors such as cognitive reserve and comorbidities in shaping clinical outcomes. However, differences persist in how these elements are applied in clinical practice. The growing availability of blood-based biomarkers and new therapeutics targeting the core pathology of AD has heightened the urgency of these discussions, particularly regarding the implications for treatment eligibility and healthcare accessibility. These evolving diagnostic frameworks underscore broader challenges in the field, including the standardization of biomarker testing, the need to account for population diversity, and the ethical considerations surrounding early detection. As AD therapeutics and research continue to advance, these criteria will likely be refined, reflecting the dynamic interplay between biological innovations and clinical application [49].

As an example of the clinical application of this recent knowledge, the Brazilian Academy of Neurology (BAN) underscores a clinically grounded approach to the use of AD biomarkers, tailored to the realities of the healthcare system. According to the BAN, biomarker testing should be reserved for symptomatic individuals with objectively verified cognitive deficits and must be conducted under the guidance of qualified specialists in Geriatrics, Neurology, or Psychiatry. The Academy advises against the use of biomarkers in asymptomatic individuals or those with subjective cognitive decline, emphasizing that their main clinical usefulness lies in supporting diagnosis for symptomatic cases [53]. This is also particularly relevant for early-onset dementia, rapidly progressive dementias, atypical clinical presentations, or when disease-modifying treatments are being considered.

The BAN supports a structured diagnostic algorithm that prioritizes CSF biomarker analysis over amyloid PET, highlighting the greater accessibility and cost-effectiveness of CSF testing in Brazil. The BAN also recognizes the potential of plasma biomarkers, provided they undergo rigorous validation to be effectively integrated into clinical practice. It emphasizes that biomarker results must be interpreted within the broader clinical context, as positive AD biomarker findings do not definitively attribute symptoms to AD pathology alone, given the high prevalence of comorbid neuropathologies in older adults [53].

## 3. Progress in Diagnostic Technologies for AD

Significant advancements in understanding the natural history of AD over the past two decades have been driven by technological breakthroughs, including the development of molecular markers for neuroimaging, biochemical analyses of biological fluids, and the implementation of bioinformatics and machine learning [54,55].

The most extensively studied biomarkers for AD are Aβ and tau proteins (Table 1). Key amyloid biomarkers include a decreased Aβ42/Aβ40 ratio in CSF and increased amyloid deposition in brain parenchyma detected in PET imaging. Additionally, elevated levels of p-tau in CSF and tau deposition in brain parenchyma in PET scans serve as effective indicators for AD pathology [56,57]. While the Aβ42/40 ratio is a validated CSF biomarker [58], it poses challenges for blood-based testing due to detection limitations [59,60]. In contrast, recent p-tau blood assays demonstrate higher diagnostic accuracy and disease specificity, making them promising candidates for routine clinical testing [61]. The p-tau217 has been demonstrated to be a superior marker of cognitive progression to dementia in patients with mild cognitive impairment (MCI) compared to p-tau181 [62]. Conversely, p-tau205 showed changes at a later stage and was more strongly associated with tau PET imaging than with amyloid PET imaging. Similar to total tau (t-tau), increased concentrations of neurofilament light chain (NfL) are not specific to AD and can also be elevated in other neurodegenerative disorders, such as amyotrophic lateral sclerosis (ALS) and frontotemporal dementia (FTD).

In a cohort of Chinese participants, changes in CSF biomarker concentrations were observed over the 20 years preceding clinical diagnosis of sporadic AD, with specific timelines as follows: Aβ42 levels changed 18 years prior, Aβ42/Aβ40 ratio 14 years prior, p-tau181 11 years prior, t-tau 10 years prior, and NfL 9 years prior to diagnosis. As cognitive impairment progressed, the alterations in CSF biomarker levels in the AD group initially accelerated before subsequently slowing down [63].

**Table 1:** Overview of diagnostic methodologies and biomarkers for AD.

Methodology	Biomarker	Advantages	Limitations	References
**Immunoassay from Biological Fluids**				
CSF	Aβ42, t-tau and p-tau, NfL	Widely used in several systematic studies	Requires the presence of cognitive decline that impacts daily activities for the diagnosis of AD	[5]
Plasma	Aβ42 and Aβ40, p-tau181, p-tau217, p-tau231, NfL, GFAP	High diagnostic accuracy for identifying AD compared to standard clinical evaluation	More research is needed	[64,65]
Serum	Aβ, tau, NfL, miRNA	Ability to accurately diagnose early stages of AD and identify individuals at high risk of cognitive decline in older adults	Diagnosis is based on clinical and pathological criteria	[66]
**Neuroimaging**				
CT	Brain atrophy and vascular changes	This method also identifies other causes of neurodegeneration	While offering ease of access, there may be less accuracy in the results	[67]
PET	Cerebral metabolism involving glycolysis or Aβ protein deposition	Identifies lower glycolysis uptake in medial temporal regions and the cingulum	High cost	[68]
MRI	Cerebral atrophy and ventricular dilation	Better visualization of atrophy in the entorhinal cortex, middle temporal lobe, and hippocampus	High cost	[67]
MRS	Measures different metabolites: NAA, mI, Chol, Glu, Gln, and GABA	Provides metabolic information that may aid in understanding AD	Difficult access and high cost of use	[69]
DTI	Description of white matter microstructure through its tensor model	Identifies potential biomarkers in the early stages of AD	Limitations regarding interpretation	[70]
FW	Isolates and quantifies changes in extracellular water	Detects subtle changes in brain tissue that may indicate early stages of DA	Introduces an additional layer of complexity in the analysis and interpretation of data	[71]

Abbreviations: NfL, neurofilament light chain; GFAP, Glial fibrillary acidic protein; miRNA, MicroRNA; MRI, magnetic resonance imaging; MRS, magnetic resonance spectroscopy; DTI, diffusion tensor imaging; FW, free-water imaging; NAA, N-acetylaspartate; mI, myo-inositol; Chol, cholesterol; Glu, glutamate; Gln, glutamine, and GABA, gamma-aminobutyric acid.

Modern molecular techniques enable the detection of Aβ40 and Aβ42 in both CSF and blood samples, with blood testing offering a less invasive alternative. Commercially available blood immunoassays for p-tau217 have demonstrated comparable accuracy to CSF biomarkers for identifying AD [65]. In a cohort study involving 786 individuals, Ashton et al. found that the p-tau217 immunoassay exhibited accuracy similar to that of cerebrospinal fluid biomarkers in identifying Aβ and tau pathologies. Plasma p-tau217 results from the ALZpath pTau217 assay were compared with magnetic resonance imaging, β-amyloid PET, tau PET, and CSF biomarkers (Aβ42/40 and p-tau immunoassays). The method was able to detect the Aβ protein across three distinct ranges in three patient cohorts. Additionally, changes were observed over an eight-year period as the disease progressed, with the most significant variation in p-tau217 noted in individuals who were positive for both Aβ and tau.

A study evaluated a two-step diagnostic workflow to screen for Aβ positivity in patients with MCI using plasma p-tau217 as a biomarker [72]. In the first step, a model combining plasma p-tau217 levels, age, and APOE ε4 status stratified patients into low-, intermediate-, and high-risk categories for Aβ-PET positivity. Confirmatory CSF testing was conducted only for intermediate-risk individuals. The workflow demonstrated high accuracy (88.2–92.0%) while reducing the need for CSF or PET confirmatory testing by 61.2–85.9%, depending on the thresholds used. The findings suggest that plasma p-tau217 can effectively aid in stratifying patients for Aβ status, potentially reducing the reliance on more invasive or costly diagnostic methods.

Advances in detection technologies, such as Single-Molecule Array (SIMOA), have enabled precise quantification of low-abundance proteins in biofluids, including Aβ peptides and p-tau, biomarkers relevant to AD. SIMOA has been employed to measure plasma Aβ42/Aβ40 ratios, providing a less invasive and more accessible method for assessing amyloid status compared to PET imaging or CSF sampling [73]. It has also been used for plasma p-tau quantification, supporting its application in early-stage AD detection [74]. The method relies on digital bead-based ELISA, where paramagnetic beads coated with capture antibodies isolate single protein molecules. Fluorescence signals are measured in femtoliter-sized wells, allowing for the detection of proteins at very low concentrations [75]. However, although SIMOA technology offers high sensitivity in biomarker detection, several limitations may impact its clinical application. Pre-analytical variability poses a significant challenge; improper sample handling, storage, or processing can lead to degradation or contamination, affecting assay accuracy. Additionally, the technology’s high sensitivity can result in the detection of clinically insignificant biomarker levels, potentially leading to false positives or overinterpretation of results. Standardization remains an issue, with limited availability of validated reference ranges and potential inter-assay variability. The technology also requires specialized equipment and trained personnel, leading to high operational costs and limiting its accessibility in resource-constrained settings. Furthermore, the assay’s performance can be influenced by factors such as reagent quality and assay design, necessitating careful optimization for each application [76,77,78,79].

Neuroimaging has proven clinically relevant for monitoring AD progression and identifying early-stage indicators. Imaging modalities such as PET, CT, magnetic resonance imaging (MRI), magnetic resonance spectroscopy (MRS), diffusion tensor imaging (DTI), and free-water imaging (FW) enable both structural and functional assessments that aid in predicting AD progression [80,81,82]. PET imaging is considered the gold standard for non-invasive AD diagnostics, playing a critical role in disease monitoring and differential diagnosis.

The development of PET radiotracers for AD has progressed through distinct phases targeting both amyloid and tau pathologies. The amyloid-targeting timeline began in 2003 with [^11^C]PiB, the first successful selective radioligand for Aβ, which led to the development and FDA approval of three ^18^F-labeled tracers: [^18^F]florbetaben (Neuraceq), [^18^F]florbetapir (Amyvid), and [^18^F]flutemetamol (Vizamyl) [83,84]. The second generation of amyloid tracers, including [^18^F]AZD4694 ([^18^F]NAV4694), addressed limitations such as white matter retention [85]. Parallel developments in tau imaging emerged with first-generation tracers such as [^18^F]THK5117 and [^11^C]PBB3, followed by [^18^F]AV-1451 (flortaucipir), which demonstrated significant binding to neurofibrillary tangles. Second-generation tau tracers, including [^18^F]MK-6240 and [^18^F]PI-2620, showed improved selectivity and reduced off-target binding to monoamine oxidases [86,87]. This dual-pathway development of both amyloid and tau radiotracers has enabled in vivo visualization of the two primary pathological hallmarks of AD, facilitating early diagnosis, disease progression monitoring, and therapeutic evaluation [88,89]. The evolution of these imaging agents has particularly enhanced the understanding of the temporal relationship between amyloid and tau accumulation in the disease process. However, the widespread clinical implementation of these modalities faces several practical challenges. These include the limited availability of radiotracers, which require proximity to a cyclotron facility due to their short half-life, as well as the considerable financial costs and infrastructure demands associated with PET imaging. These constraints affect its efficiency, diagnostic accuracy, and accessibility, particularly in routine clinical practice and in lower-income regions, where resource limitations further restrict its widespread adoption [88,89].

Additional neuroimaging techniques contribute to a comprehensive assessment in AD diagnostics. While CT imaging is less detailed than MRI, it offers a cost-effective option for initial evaluations. MRI with T1-weighted sequences provides high-resolution images, facilitating an in-depth examination of brain structures affected by AD, such as the hippocampus and entorhinal cortex [67]. MRS has demonstrated utility in detecting metabolic changes associated with AD; however, it is used less frequently due to accessibility and cost constraints [90]. DTI, a specialized form of MRI, allows for the examination of white matter microstructure, aiding in the early detection of AD by assessing neural connectivity [70]. In parallel, FW offers valuable insights by specifically detecting changes in extracellular water, highlighting early degenerative processes in the brain that conventional imaging techniques might miss [91,92].

Finally, machine learning and regression analysis represent promising tools for early AD diagnosis and predicting disease progression. Techniques such as least squares regression, support vector machines, and regression trees have been utilized to predict neuropsychological scores based on neuroimaging biomarkers [93]. Machine learning models trained on features from multiple imaging modalities have shown potential in accurately predicting cognitive outcomes, offering a compelling approach for precise diagnosis [75,81].

## 4. Are Exosomes Reliable Sources of Biomarkers for AD Diagnosis?

**Figure 1:**
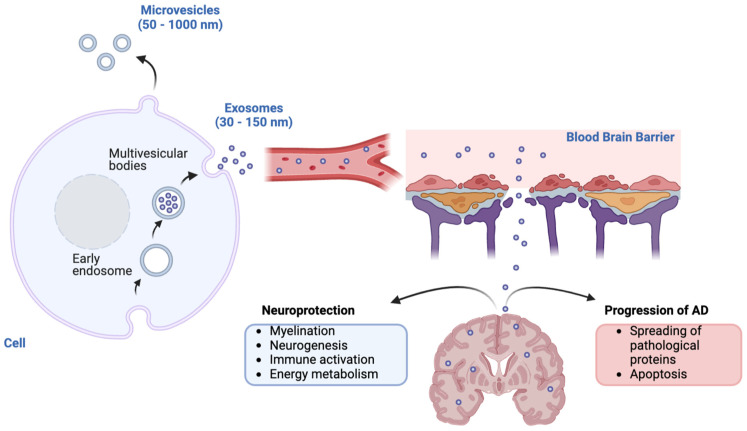
Schematic representation of the origin and function of exosomes in AD. Exosomes (30–150 nm) originate from multivesicular bodies, whereas microvesicles (50–1000 nm) bud directly from the plasma membrane. Once released into circulation, exosomes can cross the blood–brain barrier, as shown. Two main roles of exosomes in AD are depicted: (1) neuroprotection, contributing to processes such as myelination, neurogenesis, immune activation, and energy metabolism; and (2) progression of AD, mediating the spread of pathological proteins and apoptosis.

Extracellular vesicles (EVs) are lipid-coated structures produced by nearly all cell types and released into the extracellular milieu through membrane budding. They carry a variety of intracellular molecules that serve different roles within the organism, including plasma membrane restoration, intercellular communication, and the elimination of cellular waste [94,95]. The cargo and function of EVs likely contribute to their heterogeneity, leading to their classification into exosomes (30 to 150 nm) and microvesicles (50 to 1000 nm). Notably, these structures differ in their biogenesis: microvesicles are formed directly through outward budding, primarily from apoptotic cells, while exosomes are generated during the formation of endosomes (Figure 1). The endocytic pathway also provides the molecular machinery for the autophagy pathway, indicating that both exosome liberation and autophagy play crucial roles in eliminating cellular waste in a coordinated manner [96].

**Figure 2:**
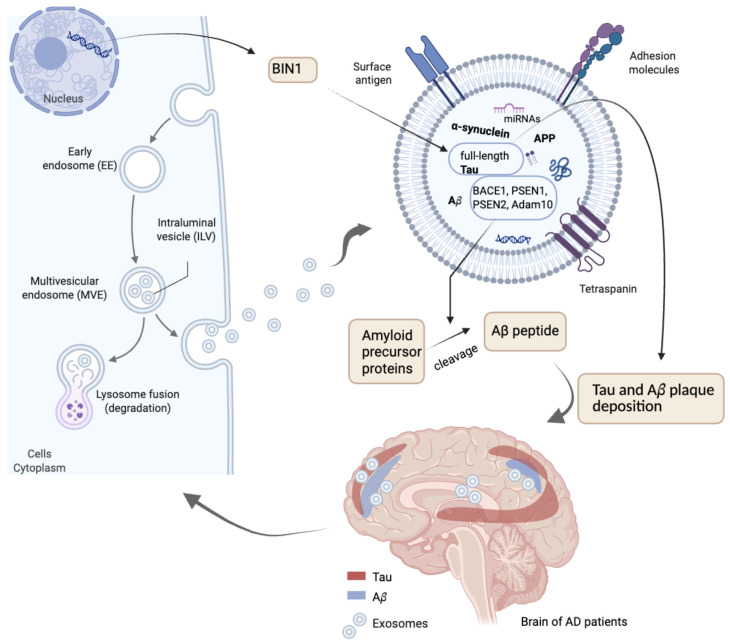
Exosomes and AD neuropathology. The figure depicts the formation of exosomes from multivesicular endosomes (MVEs) in cells and their role in AD. Exosomes carry key molecules, including amyloid precursor protein (APP), Aβ peptides, full-length tau, α-synuclein, BIN1, miRNAs, and tetraspanins, which contribute to AD pathology. These vesicles may transport molecules that participate in the cleavage of APP into Aβ peptides and, also possibly, in the deposition of tau and Aβ plaques in the brain. The bottom section highlights the distribution of tau and Aβ aggregates, alongside exosomes, in the brain of AD patients.

The role of exosomes in AD is complex, as they are implicated in both disease progression and the maintenance of normal brain physiology. Exosomes are known to be neuroprotective, supporting neuronal survival, myelination, neurogenesis, and immune responses following brain injury [97,98]. Conversely, exosomes also contribute to AD pathology by facilitating the spread of pathogenic proteins, such as Aβ and p-tau, to the brain and potentially influencing the formation of Aβ plaques (Figure 2) [99,100]. Furthermore, plasma neuron and astrocyte-derived exosomes carry some presynaptic proteins, such as neuronal pentraxin 2 (NPTX2) and neurexin 2a (NRXN2a), and their postsynaptic partners GluA4-containing glutamate (AMPA4) receptor and neuroligin 1 (NLGN1). The reduction of these molecules is related to decreased excitatory synaptic activity in the hippocampus and cerebral cortex, causing cognitive losses, as observed in AD patients [101]. Goetzl et al. quantified these four proteins in plasma neuron and astrocyte-derived exosomes of cognitively intact patients and of the same patients after AD diagnosis, 6–11 years later. They found a significative progressive decline in the levels of MPA4, NLGN1, and NRXN2a when comparing the diagnosed AD patients with those pre-diagnosed and both presented reduced levels of these molecules in relation to paired control patients that never developed AD symptoms [102].

In addition, there is evidence that the role of exosomes in AD is influenced by environmental factors, as an enriched environment induces the production of IFN-γ, which is reduced in AD patients [103]. Nakano et al. 2020 showed that in vitro treatment with IFN-γ induced the choroid plexus to produce exosomes containing miR-146a, an anti-inflammatory miRNA that is also reduced in AD patients. The increment in miR-146a possibly ameliorates astrocytic inflammation, leading to synaptogenesis and correction of cognitive impairment in these patients [104]. In addition to that, Liang et al. showed that swimming training improves the clearance of Aβ via the glymphatic system, mediated by aquaporin-4, in a mouse model of AD, with improvements in learning and memory capacity [105]. This evidence indicates that other factors such as diet, lifestyle, and exercise should modulate exosomes’ differential content and epigenetic modulations that consequently influence the progress of AD.

Due to their nanoscale size and lipid composition, exosomes can easily traverse different organs and cells, even crossing the blood–brain barrier (BBB) to interact with neurons, astrocytes, and microglia [106]. The BBB is a semipermeable multicellular complex that selectively permits the transport of specific molecules based on their physicochemical characteristics through passive diffusion or active transport and is the main entry and exit route of exosomes from the circulatory system to the brain [107,108]. Nonetheless, the precise molecular mechanism of this transport is still unclear, occurring mainly by transcytosis but also by fusion with the plasma membrane, paracytosis, and engulfment of exosomes via micropinocytosis [109]. Recently, it was shown that the receptors CD46 and integrin α5 significantly contribute to the uptake of exosomes derived from Brain-Metastatic Melanoma Cells in the BBB [110].

The glymphatic system was recently described as a system similar to the peripheral lymphatic system responsible for the clearance of interstitial fluid and waste products out of the brain through venous paravascular spaces with the participation of perivascular glial cells [111]. Furthermore, this system is impaired in AD patients, resulting in the accumulation of Aβ plaques in the brain [112]. This route has been studied to explore the potential for drug delivery and clearance, but there is still no evidence that exosomes access or leave the CNS by the glymphatic system [111]. However, it has been shown before that this system is important for the drainage of gold nanoparticles out of the brain after drug delivery [113].

In the brain, exosomes interact with the extracellular matrix, as those isolated from CSF present cytoskeletal and extracellular matrix proteins. In fact, the extracellular matrix regulates the production and capture of exosomes, while also having their remodeling regulated by exosomes’ cargo [114]. Interestingly, the extracellular matrix facilitates the engulfment of the exosomes by the target cell, remodeling their structure to promote the contact [115]. Exosomes are delivered to target cells either by fusing with the cell membrane or through endocytosis, releasing their cargo. Commonly, integrins on recipient membrane cells recognize tetraspanins (CD63 and CD9) in exosomes’ surface and allow their entry in the cell. Furthermore, proteoglycans seem to have an important role docking exosome, via linking with lectins in exosomes’ surface [116]. There is evidence that indicates a high specificity of some exosomes to interact with a specific cell type, while others can be absorbed by a larger range of cells. This is exemplified by exosomes derived from cortical neurons that can only be endocytosed by neurons’ cells, while exosomes from neuroblastoma cells interact with both neurons and glial cells [117]. After the recognition and fusion between the cell and exosomes, the cargo is released into the extracellular milieu, including molecules such as proteins, mRNA, miRNA, and circRNA. These molecules include important epigenetic modulators that have a role in gene expression regulation [118]. The contents of exosomes also reflect the pathophysiological status of the originating cells, resulting in heterogeneity in terms of size, release pathway, content, and function [94]. In the origin cell, ncRNAs are transported into the exosomes by RNA-binding proteins (RBPs), specifically by the protein heterogeneous nuclear ribonucleoprotein A2B1 (hnRNPA2B1), that recognize a sequence of nucleotides within the 5′ end of miRNAs (exo-motifs) [119]. After the binding of the RPBs and the exo-motifs, the complex is selected and transported to the forming endosome by the endosomal sorting complex required for transport (ESCRT), which ensures the proper inclusion of the ncRNAs in the exosomes [120]. Interestingly, alterations in the cellular microenvironment and stress conditions interfere in the diversity of ncRNAs present in the secreted exosomes [121].

NcRNAs, such as miRNA and circRNA, present in exosomes are increasingly being explored as potential biomarkers for various neurodegenerative diseases, including AD and chronic cognitive disorders and syndromes. Exosomes represent a promising source of biomarkers, as they are readily detectable in body fluids, particularly in serum and plasma. Furthermore, they contain specific membrane biomolecules that facilitate the identification of their originating cells. The most common exosome biomarkers are the surface tetraspanins CD63 and CD9, but Wu et al. described a technique based on a proximity barcoding assay to profile the composition of exosomes’ surface proteins and found a great diversity of proteins with a specific profile depending on the cell or tissue of origin [122]. Interestingly, exosomes derived from brain microvascular cells presented surface molecules related to their miRNA content, indicating that their cargo might regulate the distribution of exosomal surface proteins [123]. This finding provides a good perspective to detect specific exosomes in body fluid based on their membrane protein characteristics.

For a diagnostic purpose, it is possible to distinguish exosomes from different sources using commercial kits or magnetic beads linked to monoclonal antibodies, such as those for neuronal-enriched exosomes (L1 Cell Adhesion Molecule—L1CAM) and astrocyte-enriched exosomes (Glutamine aspartate transporter—GLAST) [124,125]. A recent and preliminary study demonstrated the possibility to detect differential levels of Aβ and p-tau in salivary exosomes, performing an easy isolation method using polyethylene glycol to precipitate the exosomes and Western blot analysis to quantify the protein production. With a little experimental number, Rani K et al. found a significative higher abundance of Aβ and p-tau in the salivary exosomes of AD patients in relation to other patients with cognitive impairment or control patients [126]. This is an easy and promising technique that should be more explored to find AD biomarkers.

### 4.1. Exosomal Proteins

Previous studies have demonstrated that neuronal exosomes carry a variety of misfolded proteins, including Aβ, α-synuclein, full-length APP, and p-tau [100] (Figure 2). Notably, the gene locus Bridging Integrator 1 (BIN1), which is linked to late-onset AD, has been shown to facilitate the transport of tau via exosomes both in vitro and in vivo [127]. The tau protein in exosomes represents a promising strategy for diagnostic purposes, as full-length tau has been found to be more abundant in exosomes than in the free solutions of plasma or CSF of AD patients, whereas healthy individuals do not exhibit full-length tau [128].

Exosomes also carry enzymes such as BACE1, PSEN1, PSEN2, and Adam10 (Figure 2), which are responsible for cleaving APP, generating Aβ peptides, and consequently contributing to the formation of Aβ plaques [129]. Interestingly, when peripheral blood exosomes are injected into the hippocampus of an AD mouse model, they diffuse to the cortex and exhibit affinity with microglia, clustering around Aβ plaques (Figure 2). This suggests that exosomes play a role in amyloid deposition in AD, mediated by microglia [100]. Consistently, exosomes stimulate the aggregation of Aβ1-42 in vitro. Furthermore, it has been shown that exosomes from AD patients are more neurotoxic than those from healthy individuals, impairing calcium homeostasis and mitochondrial function [130]. Additionally, the presence of exosomes is enriched in the hippocampus of AD patients compared to those with Parkinson’s disease or healthy individuals [131]. The release of exosomes is stimulated by glutamatergic activity in somato-dendritic compartments [98] and by neutral sphingomyelinase 2 (nSMase2), an enzyme that converts sphingomyelin to ceramide. Notably, inhibiting nSMase2 with GW4869 reduces exosome production, accompanied by a decrease in brain ceramide and Aβ plaque deposition [132]. Moreover, amyloid peptides induce the secretion of exosomes containing PAR-4 and C18 ceramide, which activate Caspase 3 and consequently lead to astrocyte apoptosis in vitro [133].

### 4.2. Exosomal microRNAs

miRNAs are small, double-stranded RNAs that modulate gene expression by targeting mRNA for degradation or inhibiting translation, a process known as RNA interference (RNAi) [134,135]. Several miRNAs, including miR-9, miR-29a, miR-107, miR-125b, and miR-135b, regulate various aspects of AD pathology, such as Aβ metabolism, tau phosphorylation, and neuroinflammation [136]. These miRNAs function as negative regulators of BACE1, thereby reducing Aβ production and potentially slowing the progression of AD [126].

Exosomal miRNAs are attractive biomarkers due to their stability in bodily fluids and their ability to reflect the origin and function of their originating cells. Studies have shown that miRNAs associated with AD risk genes (e.g., miR-107, miR-135b-5p) are elevated in neuronal exosomes from AD patients, while others, such as miR-320a, are reduced in the CSF of AD patients compared to healthy controls [137].

### 4.3. Exosomal Circular RNAs

CircRNAs, which are abundant in exosomes, play a crucial role in their functionality [133,138]. These stable, single-stranded RNA molecules are formed through back-splicing, resulting in a half-life exceeding 48 h [139,140,141]. They are classified into three main types: intronic circRNAs (ciRNAs), exonic circRNAs (EcircRNAs), and exon–intron circRNAs (EIciRNAs) (Isaac et al., 2021a; Titze-de-Almeida, 2023) [142,143]. EcircRNAs, which comprise over 80% of circRNAs, are derived from pre-mRNA exons and are found in both the cytoplasm and exosomes, whereas ciRNAs originate from introns and EIciRNAs are primarily located in the nucleus [143,144].

CircRNAs regulate gene expression by binding to miRNA response elements (MREs), effectively acting as sponges that sequester miRNAs from their target mRNAs [138] Additionally, circRNAs interact with proteins, influencing cellular functions and highlighting their complex regulatory roles [145,146].

Studies indicate that circRNAs can circulate through serum and are enriched in exosomes, with their levels influenced by associated miRNA variations, allowing them to transfer biological activity to target cells [138,147,148,149]. A prominent circRNA, CDR1as, exhibits high expression in the normal brain but shows reduced levels in the hippocampus and cortex of individuals with AD. CDR1-as functions as a sponge for miR-7; its decrease impairs the degradation of BACE1 and APP, leading to an abnormal increase in Aβ levels [150,151,152].

## 5. Exosomal miRNAs as Potential Biomarkers for AD Diagnosis

Recent research has focused on the role of miRNAs in AD pathogenesis associated with EVs such as exosomes (Table 2). Duan et al. examined serum-derived exosomal miRNAs in AD patients using high-throughput sequencing and RT-qPCR [153]. The authors identified hsa-miR-125b-1-3p, hsa-miR-193a-5p, hsa-miR-378a-3p, hsa-miR-378i, and hsa-miR-451a as differentially expressed miRNAs. Notably, hsa-miR-451a exhibited an area under the curve (AUC) of 0.728. Pathway enrichment analysis linked these miRNAs to neuroactive ligand–receptor interactions, PI3K-Akt signaling, and cytokine–cytokine receptor interactions [154]. Previous studies using EVs isolated from plasma samples also showed that miR-451a was significantly deregulated in AD relative to DLB [154] as well as in prodomal AD patients, highlighting its potential for differential diagnosis.

A study involving 42 AD patients and 19 healthy controls (HC) revealed significant alterations in EV membrane antigens, inflammatory cytokine content, and miRNA expression. Specifically, myelin oligodendrocyte glycoprotein (MOG) and axonal glycoprotein CD171 correlate with disease severity. Five key miRNAs (let-7g-5p, miR126-3p, miR142-3p, miR146a-5p, and miR223-3p) showed a significant reduction in AD patients, suggesting a link between disease severity and neuroinflammation [155]. Serum exosomal miR-223 levels were significantly downregulated in dementia patients compared to controls. miR-223 levels correlated with Mini-Mental State Examination (MMSE) scores and inflammatory markers (IL-1β, IL-6, TNF-α). Previous studies showed that its downregulation promotes inflammatory responses in macrophages. ROC curve analysis indicated strong diagnostic potential (AUC = 0.875) [156]. Visconte et al. found an increased expression of mR-223 in EVs from AD patients, with a positive correlation between miR-223-3p and t-tau levels, suggesting a possible involvement in the cognitive decline and neurodegeneration in AD [157].

The microRNA hsa-miR-126-3p has been identified in two distinct studies on AD. Gámez-Valero et al. found this miRNA to be significantly reduced in AD patients compared to healthy controls. Aharon et al. also observed a decrease in this miRNA in patients with severe AD compared to healthy individuals. hsa-miR-126-3p plays crucial roles in neuronal homeostasis and inflammatory response. Its reduced expression may be associated with various pathophysiological mechanisms of AD, such as neuroinflammation and apoptosis [154,155]. miR-126-3p regulates the NF-κB pathway, a key factor in the exacerbated inflammatory response observed in AD [158]. Its downregulation may contribute to a chronic neuroinflammatory environment, leading to sustained microglial activation and progressive neuronal damage.

Furthermore, studies suggest that miR-126-3p plays a fundamental role in maintaining the integrity of the BBB. Its reduced expression may increase BBB permeability, facilitating the infiltration of inflammatory cells and neurotoxic molecules into the central nervous system [159]. The decrease in miR-126-3p may also impact cellular pathways involved in protein degradation, promoting the accumulation of Aβ levels [160]. This alteration is directly linked to the formation of senile plaques and neurofibrillary tangles—hallmark features of AD. Gámez-Valero et al. suggested that the downregulation of miR-126-3p could help differentiate AD from other dementias, such as DLB. Target gene analysis revealed the regulation of pathways related to phosphorylation enzymes, the proteasome, and apoptosis, reinforcing its involvement in AD pathophysiology. However, further studies with larger cohorts and detailed functional analyses of its molecular interactions are needed to validate the clinical use of miR-126-3p as a biomarker [154].

Cha et al. observed reduced levels of miR-132 in exosomes derived from the nervous system in the plasma of AD patients compared to controls [161]. miRNA analysis of the brains of AD patients revealed lower miR-132 levels compared to high-risk pathological controls. In plasma, miR-132-3p in exosomes from the nervous system exhibited high sensitivity and specificity for diagnosing AD. miR-132 plays a key role in molecular events leading to Aβ deposition by regulating Aβ metabolism, including the tau, MAPK, and Sirtuin1 (SIRT1) pathways [162]. Another study demonstrated that miR-132/212 deficiency in an animal model resulted in exacerbated tau pathology and memory deficits, which could be partially reversed by replacement with miR-132 mimics [163]. Furthermore, reduced miR-132 expression in the hippocampus and medial frontal gyrus of AD patients (Braak scores 4 to 6) compared to non-demented controls (Braak scores 0 to 3) correlated with impairments in neuronal differentiation, mediated through the regulation of p250GAP, a Rho family GTPase enriched in the brain [164].

Recent studies have highlighted the role of miRNAs in the pathogenesis, particularly in relation to gene regulation and disease-modifying factors such as education and depression.

Wang et al. identified hsa-miR-20a-5p as a key regulator associated with AD risk in a Chinese cohort. The expression of hsa-miR-20a-5p was reduced in AD patients; however, this reduction was less pronounced in individuals with higher levels of education [165]. Notably, miR-20a-5p directly targets several genes implicated in AD, including APP, whose increased expression enhances Aβ production. The study also highlighted hsa-miR-185-5p as another downregulated miRNA in AD patients. Similar to miR-20a-5p, the decrease in hsa-miR-185-5p expression was partially offset by higher educational attainment. Functional enrichment analysis showed that validated targets of these miRNA are involved in AD-related pathways including Presenilin 1 (PSEN1), an essential component of the γ-secretase complex, which was found to be upregulated in AD patients. Additionally, the downregulation of hsa-miR-185-5p was particularly evident in AD patients with concurrent depressive symptoms. One of its predicted targets, Breast Cancer 1 (BRCA1), plays a role in PSEN1 turnover and was found to be overexpressed in AD. Collectively, these findings suggest that reduced hsa-miR-20a-5p expression contributes to increased APP levels, while the downregulation of hsa-miR-185-5p and hsa-miR-181c-5p leads to upregulation of their respective targets, facilitating Aβ accumulation and plaque formation in AD pathology [165].

The studies by Duan et al. and Kumar et al. highlight the potential role of miR-125b as a biomarker for AD [156]. hsa-miR-125b-1-3p was found to be differentially expressed in serum-derived exosomes of AD patients, showing an AUC of 0.765, with a sensitivity of 82.1%, and a specificity of 67.7%. Functional enrichment analysis indicated its involvement in key neurobiological pathways, including the PI3K-Akt and Hippo signaling pathways—an evolutionarily conserved signaling network that plays a crucial role in regulating numerous biological processes [153,166].

**Table 2:** Exosomal miRNAs in biofluids as prospective biomarkers for AD diagnosis.

EV Samples	miRNA’s	Biomarker Characteristics	References
CSF, serum, and plasma	miR-193b	Low level in CSF, serum, and plasma of AD, and serum and plasma of MCI. Potential target of the 3′ UTR of APP. Negative correlation between levels of miR-193b and Aβ42 in the CSF of patients with DAT (r = −0.442), and control group (r = −0.503).	[167]
CSF	miR-455-3p	Elevated levels in AD patients compared to controls (AUC = 0.745).	[168]
Serum	miR-223	Decreased in patients with dementia. miR-223 level correlated with Mental State Examination (MMSE) scores, Clinical Dementia Rating (CDR) scores, magnetic resonance spectroscopy (MRS) spectral ratios, and serum concentrations of IL-1b, IL-6, TNF-α, and CRP.	[169]
		miR-223 downregulated in the dementia group compared to the control group. Differential expression of miR-223 between AD and Vascular Dementia (VaD) groups. Higher miR-223 levels in AD patients under medical care than those at their first clinical visit. Levels of miR-223 in the blood of dementia patients have a positive correlation with the scores on the MMSE and CDR scales (r = 0.365 and 0.4598, respectively). miR-223 levels in patients with dementia present positive correlation with the scores on the MMSE and CDR scales (r = 0.365 and 0.4598, respectively). Levels of IL-1β, IL-6, TNF-α, and PCR elevated in patients with dementia. Higher in AD compared to VaD. A correlation was found between the levels of miR-223 and the concentrations of IL-1β, IL-6, TNF-α, and PCR (r = −0.5504, −0.4549, −0.5152, −0.4977, respectively). miR-223 present AUC of 0.875 (95% CI: 0.7779–0.9721).	
Serum	hsa-miR-125b-1-3p, hsa-miR-193a-5p, hsa-miR-378a-3p, hsa-miR-378i, and hsa-miR-451	hsa-miR-125b-1-3p has an AUC of 0.765 in the AD group compared to the healthy group. Sensitivity (82.1) and specificity (67.7%).	[147,153,170]
Plasma	miR-342-3p	Differential expression in AD group and correlated with other miRNAs decreased in AD.	[162]
	miR-185-5p, hsa-miR-20a-5p, and hsa-miR-497-5p	Related to AD and education level.	
Plasma	hsa-miR-185-5p, hsa-miR-181c-5p, hsa-miR-451a, and hsa-miR-664a-3p	Decreased hsa-miR-185-5p in AD improves the expression of PSEN1 and GSK3β, which further increases Aβ generation. The 3′ UTR of hsa-miR-181c-5p contains a predicted binding site for IL1. In AD patients, IL1 is associated with Aβ generation. hsa-miR-451a correlated with clinical measurements of education (R = 0.477), depression (R = 0.605), and leisure activity (R = 0.411). hsa-miR-664a-3p was upregulated in AD patients, which downregulated CREB1 and BDNF expression levels, thereby leading to a cognitive decline in AD patients.	[165]
Plasma	miR-16-5p, miR-19b-3p, miR-25-3p, miR-30b-5p, miR-92a-3p, and miR-451a	Validation analysis confirmed significant upregulation of miR-16-5p, miR-25-3p, miR-92a-3p, and miR-451a in prodromal AD patients, suggesting these dysregulated miRNAs are involved in the early progression of AD. Group of AD patients presented positive correlations between Aβ42 and miR-30b-5p (r = 0.67) and between h-tau and miR-223-3p (r = 0.62).	[159]
Plasma	hsa-miR-451a and hsa-miR-21-5p	Downregulated in AD samples with respect to dementia with Lewy bodies (DLB) patients.	[156]
	hsa-miR-23a-3p, hsa-miR-126-3p, hsa-let-7i-5p, and hsa-miR-151a-3p	Decreased in AD with respect to controls.	
Plasma	miR-502-5p	AUC is 0.872, sensitivity 79.2%, and specificity 83.3%.	[171]
	miR-483-5p	Area under the curve (AUC) is 0.901, sensitivity 79.2%, and specificity 100%.	
Plasma (NCAM/ABCA1 dual-labeled exosomal Aβ42/40)	miR-384	The AUC of NCAM/ABCA1 dual-labeled exosomal Aβ42/40 for diagnosis of SCD was higher than that of Aβ42, T-tau, and P-T181-tau; the AUC of NCAM/ABCA1 dual-labeled exosomal miR-384 for diagnosis of SCD was higher than that of Aβ42, Aβ42/40, T-tau, P-T181-tau, and NfL. miR-384 can downregulate the expression and activity of BACE.	[172]
Plasma (Neurons: EVL1CAM)	miR-29a-5p, miR-125b-5p, and miR-210-3p	MCI, MCI-AD, and AD dementia (AUC = 0.948).	[125]
	miR-210-3p and miR-132-5p	MCI (AUC = 0.941).	
	miR-106-5p	AD dementia (AUC = 1.000).	
	miR-106b-5p	Negative correlation with cortical thickness in regions prone to age-related dementias as imaged in MRI.	
Plasma (Astrocytes: sEVGLAST)	miR-107	MCI, MCI-AD, and AD dementia (AUCs = 0.964); AD dementia (AUC = 1.000).	
	miR-107 and miR 132-5p	Negative correlation with the cortical thickness.	
	miR-210-3p	MCI (AUCs = 0.941).	
	miR-29a-5p and miR-106-5p	Overall cognitive impairment (AUC = 0.925).	
Plasma (Microglia: sEVTMEM119)	miR-29a-5p	MCI (AUC = 0.840).	
	miR-132-5p and miR-125b-5p	AD dementia (AUC = 1.000).	
	miR-106b-5p and miR-132-5p	Negative correlation with the temporal cortical thickness.	
Plasma (Oligodendrocytes: sEVPDGFRα)	miR-29a-5p	AD dementia (AUC = 1.000). Negative correlation with temporal cortical thickness.	
Plasma (Pericytes: sEVPDGFRβ)	miR-9-5p	Overall cognitive impairment (AUC = 0.935), MCI (AUC = 0.931), and AD (AUC = 1.000).	
Plasma (Endothelial cells: sEVCD31)	miR-132-5p	Overall impairment and MCI, and prediction of AD (AUC = 1.000).	
	miR-210-3p	Negative correlation with cortical thickness.	
Plasma (Pericytes: sEVPDGFRβ and Endothelial cells: sEVCD31)	miR-9-5p (sEVPDGFRβ) and miR-132-5p (sEVCD31)	Overall cognitive impairment (AUC = 1.000).	
	miR-132-5p (sEVCD31) and miR-135b-5p (sEVPDGFRβ)	MCI and AD.	

Abbreviations: EV, extracellular vesicle; KEGG pathway, Kyoto Encyclopedia of Genes and Genomes; HC, healthy control; CSF: cerebrospinal fluid; MCI, mild cognitive impairment; 3′ UTR, 3′-untranslated region; APP, amyloid precursor protein; DAT, dementia of Alzheimer-type; PSEN1, Presenilin 1; GSK3β, Glycogen synthase kinase-3β; IL1, Interleukin-1; CREB1, cAMP-response element binding protein; BDNF, brain-derived neurotrophic factors; MMSE, Mental State Examination scores; CDR, Clinical Dementia Rating; MRS, magnetic resonance spectroscopy; TNF-α, Tumor necrosis factor alpha; VaD, Vascular Dementia; AUC, area under the curve; DLB, dementia with Lewy bodies; CI, Confidence interval; NCAM/ABCA1, neural cell adhesion molecule (NCAM)/ATP-binding cassette transporter A1 (ABCA1); SCD, subjective cognitive decline; BACE-1, beta-secretase-1.

### Critical Considerations and Constraints in Exosomal miRNA Biomarker Research for AD

Although the use of exosomes as biomarkers is very promising, their application as reliable biomarkers in AD is not without significant limitations, particularly when considered against the backdrop of challenges related to reliability and reproducibility in their isolation and disease’s inherent heterogeneity. These issues must be rigorously addressed to prevent the overinterpretation of findings and ensure meaningful application.

One of the most pressing concerns is the lack of standardization in exosome isolation protocols, which directly impacts both the purity and yield of the exosomal fractions obtained [79]. For the isolation of ncRNA from plasma or serum exosomes, all current techniques require a minimum of 500 μL of body fluid, a considerable volume, particularly for patients in weakened health. While some commercial kits enable the isolation of specific classes of exosomes or highly purified ncRNA, they often result in low yield and are very expensive. Ultracentrifugation, the most cost-effective method, offers the highest final yield but it is less pure and demands significant time and manual effort.

Studies have shown that various isolation methods can yield exosome preparations with different degrees of protein aggregates, lipoproteins, and other extracellular vesicles, complicating downstream molecular analysis and limiting cross-study comparability [78]. Additionally, biomarker identification requires large sample sizes due to significant inter-individual variability. This variability hinders the development of universal thresholds or reference ranges for exosomal biomarkers and contributes to inconsistent diagnostic performance across cohorts. Stability and storage also present critical challenges to reproducibility. A study by Yuana et al. demonstrated that long-term storage at −80 °C can preserve the exosomal structure, yet even minor deviations from protocol can impact downstream biomarker quantification [77].

Another nuanced challenge is the risk of cross-contamination and misattribution of cellular origin, especially in plasma and serum-derived exosomes. Blood contains a high abundance of exosomes from platelets, immune cells, and endothelial cells, which can mask or dilute CNS-specific vesicles. While immunoprecipitation using cell-type-specific markers (e.g., L1CAM for neuron-derived exosomes) offers a potential solution, concerns have been raised about the specificity and reliability of such markers [76].

Exosomal content is dynamic, changing with disease progression, treatment, or even daily physiological fluctuations. While this could theoretically offer insight into disease staging or treatment response, it complicates cross-sectional interpretation. Unlike imaging biomarkers that provide a relatively stable snapshot of disease burden, exosomal biomarkers require repeated sampling and careful contextualization. In the context of AD’s complex pathogenesis, the challenge is not just identifying a biomarker, but understanding how it fits into the larger mechanistic landscape. Exosomes can carry markers of Aβ, tau, inflammation, and neuronal injury—but the relative contribution of each pathway to disease onset and progression can differ across patients. This raises a critical question: Can exosomes offer more than a fragmented reflection of a multifaceted disease? Their utility may depend on integrative approaches that combine exosomal data with genomics, proteomics, imaging, and cognitive assessments to create a systems-level disease model.

## 6. Concluding Remarks and Prospects

Exosomes and their molecular contents, particularly miRNAs and circRNAs, represent a promising frontier in the search for minimally invasive and multifactorial biomarkers for AD. Their ability to encapsulate and transport molecular signals linked to key pathological processes offers a unique window into disease mechanisms and progression. Identifying specific exosomal ncRNAs associated with AD may pave the way for novel diagnostic tools that complement existing biomarkers such as Aβ and tau, ultimately refining diagnostic accuracy. However, the clinical translation of exosomal biomarkers is not without challenges. Issues such as biological heterogeneity, limited specificity, technical variability in isolation methods, and the complexity of data interpretation highlight the need for cautious optimism. Future progress will depend on rigorous methodological validation, standardization across studies, and integrative research strategies that combine exosomal insights with genomics, imaging, and clinical data. By addressing these limitations through collaborative, cross-disciplinary efforts, exosomal ncRNAs have the potential to evolve from experimental markers into reliable tools for diagnosing and understanding the pathophysiology of Alzheimer’s disease.
